# The RNA disruption assay is superior to conventional drug sensitivity assays in detecting cytotoxic drugs

**DOI:** 10.1038/s41598-020-65579-9

**Published:** 2020-05-26

**Authors:** Jonathan P. J. Mapletoft, Renée J. St-Onge, Baoqing Guo, Phillipe Butler, Twinkle J. Masilamani, Lavina D’costa, Laura B. Pritzker, Amadeo M. Parissenti

**Affiliations:** 1 Graduate Program in Chemical Sciences, Laurentian University, Sudbury, ON Canada; 2 Rna Diagnostics, Inc., Toronto and Sudbury, ON Canada; 3 Health Sciences North Research Institute, Sudbury, ON Canada; 4 Division of Medical Sciences, Northern Ontario School of Medicine, Sudbury, ON Canada

**Keywords:** Cancer, Cancer therapy, Drug development

## Abstract

Conventional drug sensitivity assays used to screen prospective anti-cancer agents for cytotoxicity monitor biological processes associated with active growth and proliferation, used as proxies of cell viability. However, these assays are unable to distinguish between growth-arrested (but otherwise viable) cells and non-viable/dead cells. As a result, compounds selected based on the results of these assays may only be cytostatic, halting or slowing tumour progression temporarily, without tumour eradication. Because agents capable of killing tumour cells (cytotoxic drugs) are likely the most promising in the clinic, there is a need for drug sensitivity assays that reliably identify cytotoxic compounds that induce cell death. We recently developed a drug sensitivity assay, called the RNA disruption assay (RDA), which measures a phenomenon associated with tumour cell death. In this study, we sought to compare our assay’s performance to that of current commonly used drug sensitivity assays (*i.e*, the clonogenic, the cell counting kit-8 and the Trypan blue exclusion assays). We found that RNA disruption occurred almost exclusively when total cell numbers decreased (cytotoxic concentrations), with little to no signal detected until cells had lost viability. In contrast, conventional assays detected a decrease in their respective drug sensitivity parameters despite cells retaining their viability, as assessed using a recovery assay. We also found that the RDA can differentiate between drug-sensitive and -resistant cells, and that it can identify agents capable of circumventing drug resistance. Taken together, our study suggests that the RDA is a superior drug discovery tool, providing a unique assessment of cell death.

## Introduction

Tumours often exhibit innate or acquired resistance to chemotherapy agents. Resistance is a major impediment to successful cancer treatment^1^, and has fuelled a search for new chemotherapy agents that kill drug-resistant tumours and/or re-establish chemotherapy sensitivity^2^. Many of the conventional drug sensitivity assays currently in use in anti-cancer drug discovery monitor a decline in cellular processes associated with healthy growing cells, including cell division, cellular metabolism and plasma membrane integrity, which are often used as proxies of cell viability. The clonogenic assay, for example, determines the fraction of drug-treated cells that retain the ability to divide and form colonies in drug-free semi-solid growth media within 1–3 weeks of treatment^3^. Another example is the cell counting kit-8 (CCK8) assay, a colorimetric assay that measures the activity of cellular dehydrogenases, representative of overall cellular metabolic activity. It quantifies the bioreduction of an exogenously supplied disulfonated tetrazolium salt into an orange formazan product^4,5^. The Trypan blue exclusion assay is also used in drug discovery. Relying on the observation that certain cell-impermeable dyes can only traverse compromised plasma membranes, usually encountered in dead cells, the Trypan blue exclusion assay can be used to assess the effects of a drug on cell viability by monitoring the loss of membrane integrity^6^. Though routinely used as so-called ‘viability’ assays, it is important to note that the relationship between the assays’ drug sensitivity parameter (*i.e*., cell division, cellular metabolism, membrane integrity) and true viability is not always supported. For example, the clonogenic assay was found to be unable to distinguish between arrested (but viable) cells and dead cells^7^, and the CCK8 assay responds to changes in metabolic activity, irrespective of whether these changes reflect a loss of viability^8^. Cells that have lost membrane integrity are not necessarily dying or dead^9^, and cells that have intact membranes are not necessarily viable^10,11^. Because anti-cancer drugs capable of killing tumour cells, as opposed to merely inhibiting their growth, are likely to show the most promise in the clinic, there is a need for drug sensitivity assays that can more effectively discriminate between viable cells (including actively growing cells and growth-arrested cells with the potential to resume growth) and non-viable (dead) cells.

Our research group developed a drug sensitivity assay called the RNA disruption assay (RDA)^12^, which monitors a biological process associated with tumour cell death, rather than parameters of active proliferating cells^13^. The process, which is triggered by a wide variety of chemotherapy agents, is characterized by the degradation of ribosomal RNAs (rRNAs) into long-lived high-molecular-weight fragments^12,13^. As the resulting RNA profile is quite distinct from that generated during autolytic RNA degradation, the phenomenon was termed ‘RNA disruption’^12^. The RDA was developed to specifically quantify the phenomenon. The assay’s metric, the RNA disruption index (RDI), is calculated using a proprietary algorithm and is essentially the ratio between abnormal and normal rRNAs. It is thus directly proportional to the degree of chemotherapy-induced RNA disruption^12^.

RNA disruption can be demonstrated *in vitro* in many cancer cell lines from different tissues and in response to multiple, structurally distinct chemotherapy agents with different mechanisms of action^13^, suggesting that many pathways activated by anti-cancer drugs culminate in RNA disruption. RNA disruption itself may accompany and/or contribute to tumour cell death, as it is temporally associated with the induction of apoptosis in docetaxel-treated ovarian cancer cells, and a caspase-3 inhibitor attenuates drug-induced RNA disruption^13^. RNA disruption may not be restricted to apoptosis-mediated cell death, as rRNA cleavage has been shown to occur in the absence of apoptosis^14^.

Chemotherapy-induced RNA disruption was also observed by Parissenti *et al.*^12^ and Pritzker *et al.*^15^ in breast tumours of women with locally advanced or inflammatory breast cancer who had been treated with a combination of epirubicin and docetaxel in a sub-study of the NCIC-CTG-MA.22 clinical trial^16^. Consistent with the *in vitro* studies, RNA disruption was associated with a loss of tumour cell viability *in vivo*, as extensive RNA disruption within tumours during treatment was positively associated with pathological complete response and enhanced disease-free survival in patients post-treatment^12,15^. During the TCHL (NCT01485926) clinical trial, Toomey *et al.*^17^ noted a similar relationship between RNA disruption and pathological complete response in women with human epidermal growth factor receptor 2-positive breast cancer treated with a combination of docetaxel, carboplatin and trastuzumab, with or without lapatinib.

Because the RDA uniquely measures a biological process specifically triggered by a wide variety of anti-cancer drugs, and because RNA disruption is associated with the loss of tumour cell viability both *in vitro* and *in vivo*, the RDA may be a useful tool for discovering new and highly effective anti-cancer agents. In this study, we sought to determine whether the RDA can discriminate between viable (proliferating or growth-arrested) cells and non-viable cells after drug exposure, and to compare its performance to that of conventional drug sensitivity assays. We also explored the RDA’s ability to differentiate between drug-sensitive and -resistant cells, and to identify bioactive compounds capable of restoring sensitivity to drug-resistant cells.

## Results

### The RDA discriminates between viable and non-viable drug-treated cells

The most promising anti-cancer drugs are capable of killing tumour cells *in vivo*; merely inhibiting their proliferation could result in disease progression or recurrence upon treatment completion. Since RNA disruption occurs in response to a wide variety of chemotherapeutic drugs with different mechanisms of action^13^, and because it is positively associated with tumour cell death *in vitro*^13^ and *in vivo*^12,15^, we hypothesized that the RDA could discriminate between cytotoxic (death-inducing) and non-toxic drugs (or drug doses), and be a powerful tool in anti-cancer drug discovery efforts.

**Figure 1:**
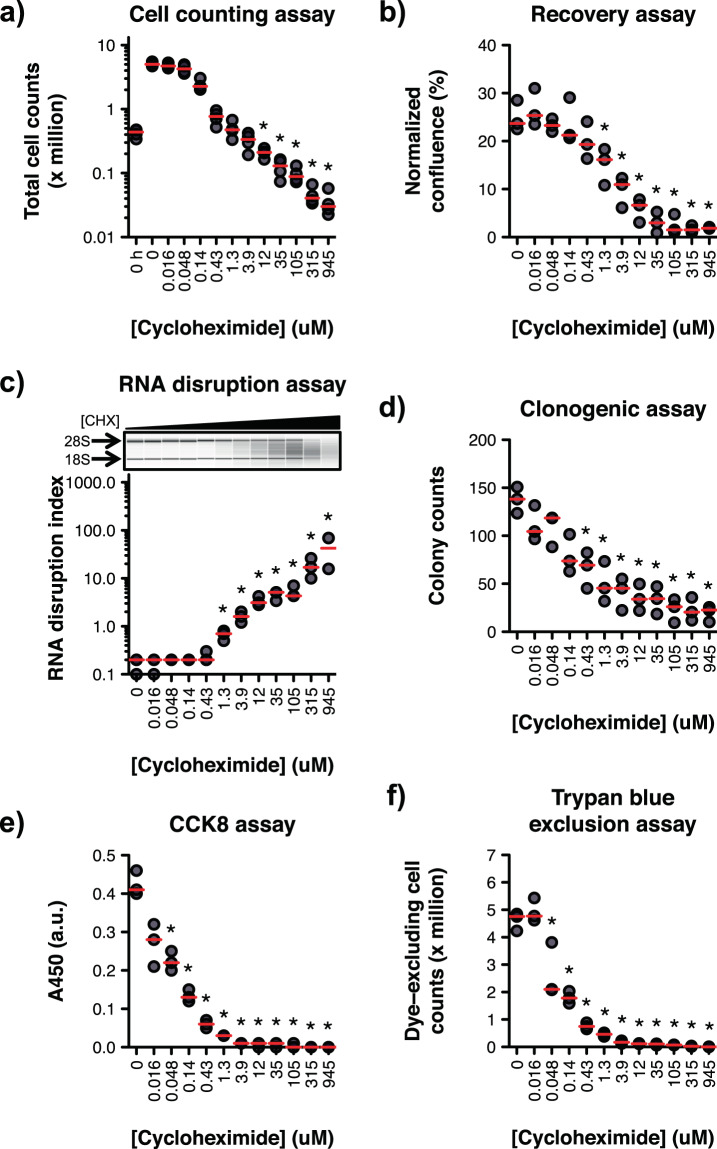
Comparison of the ability of drug sensitivity assays to discriminate between viable and non-viable cycloheximide-treated cells. **(a)** A2780 cells were treated with cycloheximide for 72 h, and total cells were counted using a haemocytometer. Untreated cells were also counted prior to plating, at 0 h. Kruskal-Wallis test, *χ*^2^(12) = 49.68, *n* = 4, *P* < 0.001. **(b)** A2780 cells were treated with cycloheximide for 72 h. Cells were then collected, resuspended in drug-free medium, and seeded into plates. After 96 h, culture confluence was measured and normalized to the initial confluence. The time-course for a representative replicate is depicted in Supplementary Fig. S1. Kruskal-Wallis test, *χ*^2^(11) = 31.47, *n* = 3, *P* < 0.001. **(c)** Total RNA was isolated from A2780 cells treated with cycloheximide (CHX) for 72 h, and size-separated by capillary gel electrophoresis. The degree of RNA disruption was then quantified using the RDA. A representative electropherogram (cropped for clarity) is shown. Full-length 18 S and 28 S rRNA bands are indicated with arrows. The full-length electropherogram is provided in Supplementary Fig. S2. Kruskal-Wallis test, *χ*^2^(11) = 32.68, *n* = 2*–*3, *P* < 0.001. **(d)** A2780 cells were treated with cycloheximide for 24 h, and proliferating cells remaining post-treatment were counted using the clonogenic assay. Colony counts indicate the average number of colonies per field of view. Kruskal-Wallis test, *χ*^2^(11) = 28.71, *n* = 3, *P* = 0.003. **(e)** A2780 cells were treated with cycloheximide for 72 h, and cellular dehydrogenase activity was quantified using the CCK8 assay. Kruskal-Wallis test, *χ*^2^(11) = 33.31, *n* = 3, *P* < 0.001. a.u., arbitrary units. **(f)** A2780 cells were treated with cycloheximide for 72 h. Cells were then stained with Trypan blue, and dye-excluding cells in each plate well were counted using a haemocytometer. Kruskal-Wallis test, *χ*^2^(11) = 34.45, *n* = 3, *P* < 0.001. Red dashes indicate the group median. Groups annotated with an asterisk are significantly lower (panels a, b, d, e and f) or greater (panel c) than the ‘0 h’ group (panel a) or the ‘0 μM’ group (panels b, c, d, e and f).

We began our investigation by determining whether our assay was capable of distinguishing between viable cells (proliferating or intact growth-arrested cells) and non-viable/dead cells following treatment with cycloheximide, a drug known to be non-cytotoxic at low doses and cytotoxic at high doses^18^. To define the range of cycloheximide concentrations deemed cytotoxic and non-cytotoxic in our system, we first treated ovarian cancer cells with or without increasing concentrations of cycloheximide, and monitored the loss of cell viability by quantifying the total number of cells remaining post-treatment using a haemocytometer. To confirm lethality, and the irreversible commitment to cell death, we coupled our cell count experiment with a recovery assay, in which treated cells were allowed to grow post-treatment for several days in a nutrient-rich, drug-free medium. In this work, drugs and drug concentrations were deemed cytotoxic if they decreased total cell numbers during treatment. We found that cycloheximide concentrations ≥ 12 μM significantly reduced total cell numbers during treatment (Fig. 1a), suggesting that cycloheximide concentrations ≥ 12 μM were cytotoxic. Compromised cell recovery from drug treatment, owing to cell death or continued cell cycle arrest, was achieved at cycloheximide concentrations ≥ 1.3 μM (Fig. 1b, Supplementary Fig. S1).

To test whether the RDA was capable of discriminating between viable and non-viable drug-treated cells, we next exposed our cells to both cytotoxic (≥ 12 μM) and non-cytotoxic (<12 μM) concentrations of cycloheximide, and then probed the cells for the accumulation of RNA disruption fragments using the RDA. We detected significant RNA disruption only in cell populations exposed to drug concentrations that hamper post-treatment recovery (≥ 1.3 μM), and populations treated with cytotoxic drug concentrations (≥ 12 μM) (Fig. 1c, Supplementary Fig. S2), suggesting that the RDA showed responsiveness almost exclusively to cells that have lost their viability, and little to no responsiveness to viable cells.

### The RDA is more discriminating of viable and non-viable drug-treated cells than classical drug sensitivity assays

Conventional drug sensitivity assays monitor biological processes associated with healthy growing cells (*e.g*., cell replication, metabolism and plasma membrane integrity), and are thus likely to misidentify growth-arrested (but viable) cells as dead cells, because arrested cells do not replicate. Since extensive RNA disruption was seen in non-viable cells, we sought to compare the RDA’s performance to that of other drug sensitivity assays. We selected three widely used drug sensitivity assays that monitor different cellular phenotypes: the clonogenic assay (cell replication and colony formation), the CCK8 assay (cellular metabolism) and the Trypan blue exclusion assay (plasma membrane integrity). We monitored these phenotypes in ovarian cancer cells treated with both cytotoxic and non-cytotoxic concentrations of cycloheximide. We found that the clonogenic, CCK8 and Trypan blue exclusion assays detected a significant decrease in colony formation (Fig. 1d), cellular metabolism (Fig. 1e) and plasma membrane integrity (Fig. 1f), respectively, in cells treated with cycloheximide doses (0.048–0.43 μM) that did not impact cell viability and from which treated cells readily recovered replicative capacity upon drug removal (Fig. 1a and 1b, Supplementary Fig. S1). Comparatively, such non-cytotoxic drug concentrations did not trigger significant RNA disruption in cells; a ~3- to 27-fold increase in drug concentration was necessary for RNA disruption to occur (Fig. 1c, Supplementary Fig. S2). Taken together, our results suggested that the RDA detected dead cells more effectively than current drug sensitivity assays.

### The RDA differentiates between drug-sensitive and drug-resistant cells

**Figure 2:**
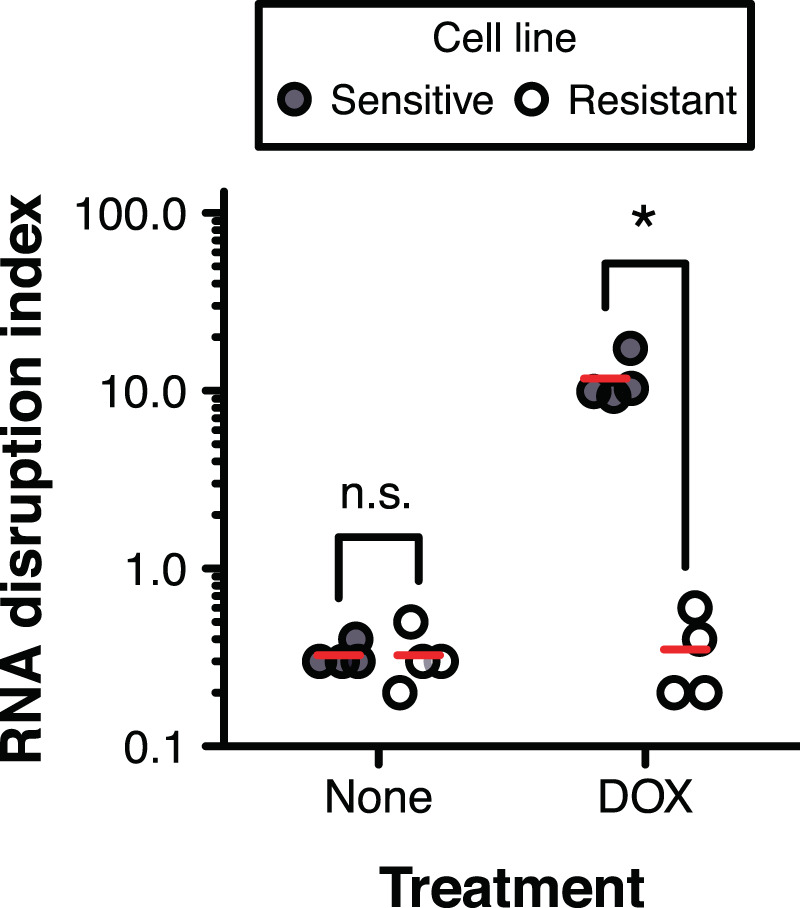
Doxorubicin-induced RNA disruption in drug-sensitive and -resistant cells. Doxorubicin-sensitive A2780 cells (Sensitive) and doxorubicin-resistant A2780_ADR_ cells (Resistant) were treated for 72 h with 0 or 500 nM doxorubicin (DOX). Total RNA was extracted from cells, size-separated by capillary gel electrophoresis, and the level of RNA disruption was measured using the RDA. A two-way ANOVA with drug resistance (sensitive, resistant) and treatment (none, DOX) as between-subjects factors found a significant interaction between drug resistance and treatment [*F*(1, 12) = 93.24, *n* = 4, *P* < 0.001]. Red dashes indicate the group mean. Group pairs annotated with an asterisk are significantly different. Only comparisons between cell lines at each treatment level are shown. n.s., non-significant.

Given the greater discriminatory power of the RDA over existing drug sensitivity assays to detect non-viable cells, we next sought to test the performance of the RDA in a drug discovery context by determining whether the RDA could be used to identify drugs that restore sensitivity to resistant cells. We launched our investigation by first establishing whether the RDA can distinguish between doxorubicin-sensitive and doxorubicin-resistant ovarian cancer cells. We treated drug-sensitive and -resistant cells for 72 h with 500 nM doxorubicin, and monitored RNA disruption in treated cells using the RDA. We found that the RDIs of drug-treated sensitive cells were consistently high, whereas the RDIs of drug-treated resistant cells were significantly lower and comparable to those of untreated drug-sensitive and -resistant cells (Fig. 2).

**Figure 3:**
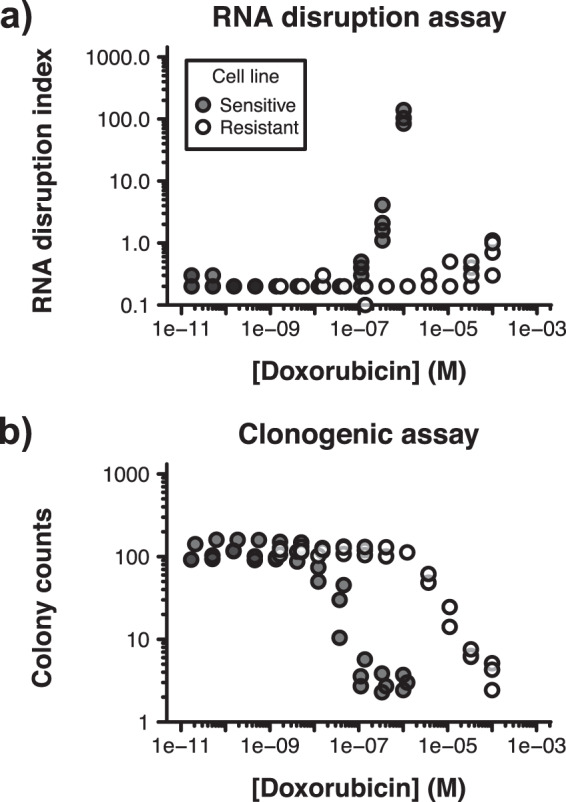
Discrimination between drug-sensitive and drug-resistant cells using the RDA and the clonogenic assay. Doxorubicin-sensitive A2780 cells (Sensitive) and doxorubicin-resistant A2780_ADR_ cells (Resistant) were treated with a wide range of doxorubicin concentrations for 72 h (RDA) or 24 h (clonogenic assay). **(a)** Total RNA was extracted from cells, size-separated by capillary gel electrophoresis, and the extent of RNA disruption was scored using the RDA. Representative electropherograms are shown in Supplementary Fig. S3. *n* = 3–4. **(b)** Proliferating cells remaining post-treatment were counted using the clonogenic assay. *n* = 3.

To complement our findings, we also monitored RNA disruption in doxorubicin-sensitive and -resistant cells upon treating cells with a wide range of doxorubicin concentrations for 72 h. We found that greater levels of doxorubicin were required to cause RNA disruption in doxorubicin-resistant cells than in doxorubicin-sensitive cells (Fig. 3a, Supplementary Fig. S3). Our findings suggested that the RDA readily differentiated between drug-sensitive and drug-resistant cells (based on the extent of RNA disruption occurring in response to treatment).

Additionally, we were interested in comparing the RDA’s performance to that of the gold standard of drug sensitivity assays (the clonogenic assay). To this end, we monitored colony formation by sensitive and resistant cells after treatment for 24 h with increasing concentrations of doxorubicin and subsequent introduction into drug-free medium. Similarly to what we observed with the RDA, we found that higher doses of doxorubicin were needed to reduce colony formation in drug-resistant cells compared to drug-sensitive cells (Fig. 3b). Taken together with our earlier observations, our results suggested that the RDA performed comparably to the clonogenic assay with respect to differentiating between drug-sensitive and -resistant cells.

### The RDA enables the identification of compounds that restore sensitivity to drug-resistant cells

Having established the ability of the RDA to discriminate between drug-sensitive and drug-resistant cells, we next tested the ability of the RDA to identify bioactive molecules that can restore sensitivity to drug-resistant cells. We first sought to identify the major cellular mechanism conferring resistance to the doxorubicin-resistant cells, with the ultimate intention of using small molecules to circumvent this resistance mechanism and then monitoring the restoration of drug sensitivity using the RDA.

**Figure 4:**
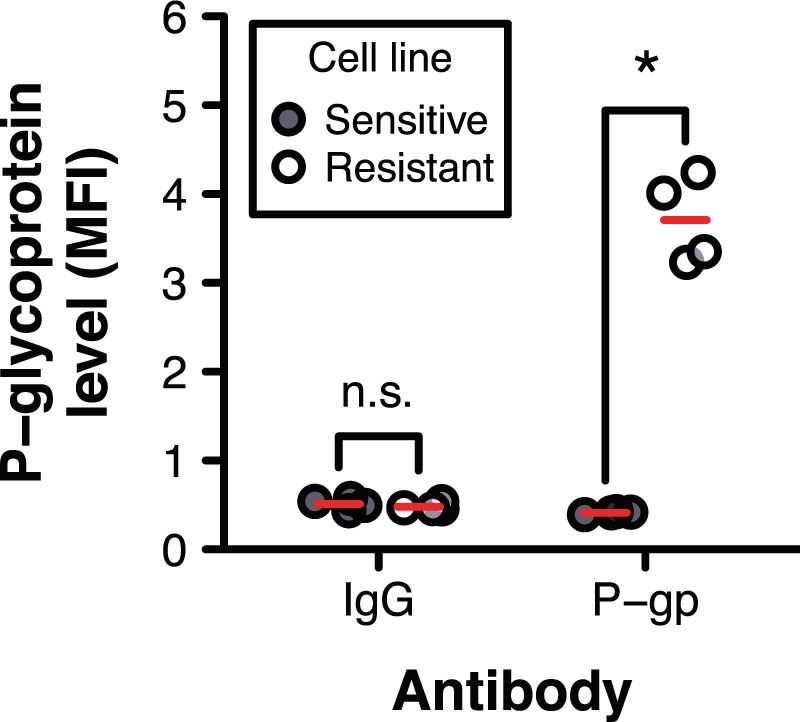
Cell surface-associated P-gp protein expression in doxorubicin-sensitive and -resistant cells. Surface expression of P-gp in doxorubicin-sensitive A2780 cells (Sensitive) and doxorubicin-resistant A2780_ADR_ cells (Resistant) was assessed by flow cytometry using an R-phycoerythrin-conjugated mouse anti-human P-gp antibody (P-gp). Flow cytometry using an R-phycoerythrin-conjugated mouse IgG2b κ isotype antibody (IgG) was also performed in parallel, as a negative control. A two-way ANOVA with drug resistance (sensitive, resistant) and the antibody used during flow cytometry (IgG, P-gp) as between-subjects factors identified a significant interaction between drug resistance and the antibody used [*F*(1, 12) = 514.82, *n* = 4, *P* < 0.001]. Red dashes indicate the group mean. Group pairs annotated with an asterisk are significantly different. Only comparisons between cell lines at each antibody level are shown. A representative flow cytometry graph is shown in Supplementary Fig. S4. MFI, mean fluorescence intensity, in relative fluorescence units.

**Figure 5:**
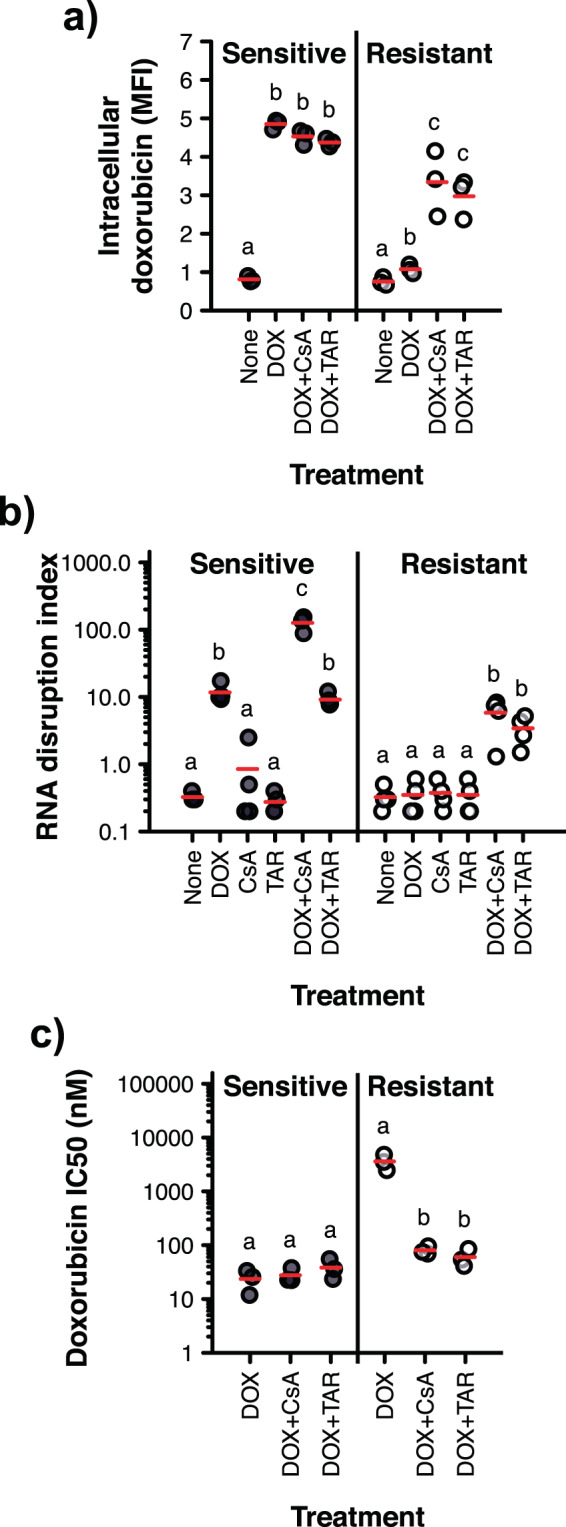
Effect of P-gp inhibition on intracellular drug accumulation, RNA disruption and cell proliferation. **(a)** Doxorubicin-sensitive A2780 cells (Sensitive) and doxorubicin-resistant A2780_ADR_ cells (Resistant) were treated for 24 h with 0 or 500 nM doxorubicin (DOX), in the presence or absence of 5 μM CsA or 100 nM TAR. Intracellular doxorubicin levels were then quantified by flow cytometry. A two-way ANOVA with drug resistance (sensitive, resistant) and treatment used (none, DOX, DOX + CsA, DOX + TAR) as between-subjects factors revealed a significant interaction between drug resistance and treatment [*F*(3, 16) = 33.13, *n* = 3, *P* < 0.001]. MFI, mean fluorescence intensity, in relative fluorescence units**. (b)** Doxorubicin-sensitive A2780 cells (Sensitive) and doxorubicin-resistant A2780_ADR_ cells (Resistant) were treated for 72 h with 0 or 500 nM doxorubicin (DOX), in the presence or absence of 5 μM CsA or 100 nM TAR. Total RNA was extracted from cells, size-separated by capillary gel electrophoresis, and the level of RNA disruption was measured using the RDA. A two-way ANOVA with drug resistance (sensitive, resistant) and treatment (none, CsA, TAR, DOX, DOX + CsA, DOX + TAR) as between-subjects factors found a significant interaction between drug resistance and treatment [*F*(5, 35) = 16.57, *n* = 3–4, *P* < 0.001]. **(c)** Doxorubicin-sensitive A2780 cells (Sensitive) and doxorubicin-resistant A2780_ADR_ cells (Resistant) were treated for 24 h with a wide range of doxorubicin (DOX) concentrations, in the absence or in the presence of either 5 μM CsA or 100 nM TAR. Proliferating cells remaining post-treatment were then counted using the clonogenic assay. The concentration of doxorubicin at which the survival fraction is reduced by 50% (IC50) was calculated. A two-way ANOVA with drug resistance (sensitive, resistant) and treatment (DOX, DOX + CsA, DOX + TAR) as between-subjects factors found a significant interaction between drug resistance and treatment [*F*(2, 12) = 68.09, *n* = 3*, P* < 0.001]. Representative flow cytometry graphs, electropherograms and survival curves are provided in Supplementary Fig. S5, S6 and S7. Red dashes indicate the group mean. For a given cell line, group pairs annotated with the same letter are not significantly different. Only comparisons between treatments at each cell line level are shown.

One common mechanism of acquired drug resistance involves the induction of ATP-binding cassette transporters, such as P-glycoprotein (P-gp)^19^, which promote the efflux of chemotherapy agents from tumour cells^20^. For example, previous studies have shown that transcripts encoding P-gp, a drug efflux transporter known to promote docetaxel efflux from cells^21^, were more abundant in docetaxel-resistant cells than in docetaxel-sensitive cells^22^. We therefore postulated that P-gp proteins are highly overexpressed in the doxorubicin-resistant cells used herein, in comparison to sensitive cells, leading to greater doxorubicin efflux and decreased sensitivity. We quantified cell surface-associated P-gp proteins in untreated doxorubicin-sensitive and -resistant cells using flow cytometry, and discovered high levels of P-gp proteins on the surfaces of drug-resistant cells compared to drug-sensitive cells (Fig. 4, Supplementary Fig. S4). To determine if P-gp overexpression coincided with decreased drug abundance within cells, we treated sensitive and resistant cells with intrinsically fluorescent doxorubicin, and measured the intracellular drug levels using flow cytometry. We observed lower levels of doxorubicin within resistant cells than within sensitive cells (Fig. 5a, Supplementary Fig. S5), suggesting that doxorubicin resistance in the resistant cell line may be attributed, at least in part, to P-gp overexpression.

Having demonstrated that P-gp likely plays a role in doxorubicin resistance in the cell line used in this study, we next sought to monitor the loss of resistance (*i.e*., the increase in cell death) of drug-resistant cells upon restoring their sensitivity to doxorubicin through P-gp inhibition. We used two known inhibitors of P-gp activity: (i) cyclosporine A (CsA), a pan competitive inhibitor of ATP-binding cassette transporters^23^, and (ii) tariquidar (TAR), an inhibitor known to be highly specific for P-gp at low concentrations^24–26^. We found that exposing doxorubicin-treated resistant cells to either CsA or TAR significantly increased intracellular doxorubicin levels, with levels nearing those in doxorubicin-treated sensitive cells (with or without inhibitor) (Fig. 5a, Supplementary Fig. S5). This increase in intracellular drug levels coincided with a significant increase in RNA disruption, with RDIs comparable to those of drug-treated sensitive cells (without inhibitor) (Fig. 5b, Supplementary Fig. S6). Interestingly, we also noted that CsA (but not TAR) significantly increased the sensitivity of the sensitive cells to doxorubicin (Fig. 5b, Supplementary Fig. S6), even though (i) P-gp protein expression was found to be negligible in the sensitive cells (Fig. 4, Supplementary Fig. S4), and (ii) CsA did not significantly impact intracellular doxorubicin levels in treated sensitive cells (Fig. 5a, Supplementary Fig. S5). The noted increased sensitivity may be the result of CsA’s impact on cellular targets other than P-gp. For example, CsA binds calpain-2, caspase-3 and p38 mitogen-activated protein kinase 14 (three enzymes that play a role in the regulation of apoptosis), and CsA increases their activity in *in vitro* assays^27^. CsA has previously been shown to induce caspase-3- and -9-dependent apoptotic cell death in human lung adenocarcinoma cells^28^. Interestingly, caspase-3 may also play a role in drug-mediated RNA disruption^13^.

Again, we compared the performance of the RDA to that of the gold standard of drug sensitivity assays by monitoring colony formation in doxorubicin-treated sensitive and resistant cells in the presence or absence of P-gp inhibitors using the clonogenic assay. Consistent with our RNA disruption findings, we found that CsA and TAR significantly increased doxorubicin sensitivity in the drug-resistant cells, with the doxorubicin half-maximal inhibitory concentration (IC50) of the inhibitor-treated cells decreasing to levels similar to those of drug-treated sensitive cells, in the presence or absence of inhibitor (Fig. 5c, Supplementary Fig. S7). These observations were consistent with the inhibitor-mediated increase in intracellular doxorubicin levels (Fig. 5a, Supplementary Fig. S5).

Taken together, our findings suggested that, like conventional drug sensitivity assays, the RDA could be used to monitor small molecule-induced changes in the drug sensitivity of tumour cells.

## Discussion

Conventional drug sensitivity assays measure parameters associated with viable tumour cells, including ‘unlimited’ cell division (clonogenic assay), robust metabolism (CCK8 assay) and intact plasma membranes (Trypan blue exclusion assay). A reduction in any of these parameters is considered to signal a reduction in cell viability. However, earlier studies have shown that the relationship between the drug sensitivity parameter measured by the assay and true viability is imperfect. For example, Waldman *et al.*^7^ found that the clonogenic assay detected no change in the sensitivity of colorectal cancer cells to γ-radiation upon compromising their p21-dependent cell cycle checkpoint. However, irradiated p21-proficient cell line cultures contained considerably more arrested (viable) cells than p21-deficient cell line cultures (as determined by measuring cell adherence), suggesting that the clonogenic assay is unable to discriminate between arrested and dead cells^7^. Tran *et al.*^9^ noted that the Trypan blue exclusion assay suffered from similar limitations. They found that exposure of murine macrophages to a low dose of the membrane pore-forming haemolysin HlyII from *Bacillus cereus* did not dramatically impact the cells’ metabolic activity (as determined using the MTS and ATP assays)^9^. Cells were also able to repair their HlyII-injured membranes during a 24-h recovery period following treatment^9^. However, though cells were clearly viable, they nevertheless took up the vital dye Trypan blue, demonstrating that the Trypan blue exclusion assay can misidentify viable cells as dead^9^. The Trypan blue exclusion assay can also misidentify dying/dead cells as viable cells. For example, mouse lymphoma cells treated with either mitomycin C, colchicine or carbendazim are non-viable (as assessed by cloning efficiency and total growth) and yet, they nevertheless exclude Trypan blue^10^.

Given the limitations of these current and long-standing methodologies, we explored the utility of the RDA as an alternative method to existing drug sensitivity assays. In this study, we found that the RDA readily distinguishes between viable cells and dying/dead cells. RNA disruption was detected almost exclusively in ovarian cancer cells treated with a lethal dose of cycloheximide; little to no disruption was measured in cells treated with a non-cytotoxic concentration of the drug (Fig. 1). In stark contrast to these observations, the clonogenic, CCK8 and Trypan blue exclusion assays detected a reduction in colony formation, cellular metabolism and membrane integrity, respectively − traditionally interpreted as indicating a loss of viability − in cells exposed to non-cytotoxic doses of cycloheximide (Fig. 1). Our results suggest that the RDA responds almost exclusively to cytotoxic drug concentrations, and may thus prove to be a superior and more robust drug discovery assay when seeking to identify agents that promote cell death. Furthermore, the RDA offers many advantages with respect to its implementation in the laboratory (Supplementary Table S1). For example, the RDA involves relatively few steps, is amenable to automation using automated liquid handlers, is high-throughput when automated, and can be completed within a single day. The clonogenic assay, in contrast, is very labour-intensive and time-consuming as scoring requires colony formation, which can take up to three weeks, depending on the cell line. The clonogenic assay is also more prone to human error, particularly when colonies are counted manually, as there is some subjectivity in terms of what constitutes a positive colony. The Trypan blue exclusion assay is not high-throughput, and it is also subject to human error (associated with cell dispersion and dilution). Furthermore, the dye is toxic to *in vitro* cultured mammalian cells, causing a reduction in total cell numbers while cell counting is still in progress^29^. The implementation of the RDA is, however, more costly than other drug sensitivity assays currently available, owing to the need for specialized instrumentation, reagents and consumables.

We also investigated the utility of the RDA to identify bioactive molecules capable of restoring sensitivity to drug-resistant tumour cells. As a proof-of-concept, we monitored the drug sensitivity of doxorubicin-resistant cells upon attenuating their major resistance mechanism. Having found that (i) a drug efflux pump, P-gp, is overexpressed in the doxorubicin-resistant cells (Fig. 4, Supplementary Fig. S4), and that (ii) this is accompanied by relatively low intracellular drug levels (Fig. 5a, Supplementary Fig. S5), we reduced P-gp activity in resistant cells using known P-gp inhibitors (CsA and TAR) and monitored cell death using the RDA. The increase in intracellular doxorubicin levels observed in resistant cells upon treatment with a P-gp inhibitor (Fig. 5a, Supplementary Fig. S5) coincided with the initiation of RNA disruption, with disruption levels nearing those seen in drug-treated sensitive cells (Fig. 5b, Supplementary Fig. S6). The RDA successfully reflected the restoration of drug sensitivity in resistant cells in which the resistance mechanism was compromised, demonstrating that the RDA could be used as a tool to discover drug-sensitizing agents for chemoresistant cells. Results obtained using the RDA were mirrored by those obtained using the clonogenic assay (Fig. 5c, Supplementary Fig. S7).

Previous studies in our laboratory have shown that RNA disruption occurs in both floating cells and adherent cells after drug treatment, with the number of floating cells increasing with increased RNA disruption. RNA disruption was found to be higher in floating cells than in adherent cells (unpublished observations). It is possible that drug-induced ribosome fragments from floating cells are harvested with trypsin-released adherent cells during the centrifugation step used to collect all drug-treated cells. However, we have also observed that all cells harvested post-treatment exhibit a sub-G1 DNA content, confirming that the vast majority of adherent cells are non-viable after drug-treatment (Butler *et al*., manuscript in preparation). Likely, RNA disruption precedes loss of cell adhesion and loss of cell integrity.

Because a variety of structurally and mechanistically distinct stressors, including various chemotherapy agents, induce RNA disruption in different cancer cell lines, and because RNA disruption is consistently associated with loss of cell viability^13^ (Butler *et al*., manuscript in preparation), the RDA can be broadly applied to monitor cell death in different cell types and in response to different anti-cancer drugs. We have observed, however, that drugs vary in the extent of RNA disruption that they induce (Butler *et al*., manuscript in preparation). As a result, there is the prospect that the RDA, by itself, may overlook interesting compounds that, while lethal to the cell, trigger only minimal RNA disruption. Thus, coupling the RDA with other assays that monitor death-related processes (such as flow cytometric detection of drug-treated cells with a sub-G1 DNA content) will remain useful to identify cell death-inducing agents in anti-cancer drug discovery programs.

## Methods

### Ethics approval

This study did not require ethics approval from an ethics review committee because the study did not involve animals, humans, human data or material collected directly from animals or humans.

### Cell culturing

The A2780 ovarian cancer cell line and its doxorubicin-resistant variant, A2780_ADR_, were purchased from the European Collection of Authenticated Cell Cultures. Unless otherwise indicated, both cell lines were cultured in 75-cm^2^ vented tissue culture flasks (Corning) in L-glutamine-containing RPMI 1640 medium (GE Healthcare Life Sciences) supplemented with 10% foetal bovine serum (FBS) (Gibco). Cultures were maintained in a humidified incubator at 37 °C in 5% CO_2_, and passaged every 2–3 days. A2780_ADR_ cells were maintained in growth medium supplemented with 100 nM doxorubicin (Accord). Before performing experiments, A2780_ADR_ cells were incubated in drug-free medium for at least 4 days to minimize acute drug effects. Cell lines were passaged no more than 30 times. All cell lines were confirmed to be free of *Mycoplasma* using the PCR Mycoplasma Detection kit (Applied Biological Materials).

### Cell counting and recovery assays

Cells were seeded in a 6-well flat-bottom tissue culture plate (Sarstedt) at a density of 150,000 cells per well in 3 mL drug-free medium. The plate was incubated in a humidified incubator at 37 °C with 5% CO_2_ for 24 h. After 24 h, the medium was replaced with 3 mL fresh drug-free medium or fresh medium supplemented with ≤945 µM cycloheximide (Sigma-Aldrich), and the plate was incubated for an additional 72 h as described above. The medium (containing floating cells) was then transferred to a 15-mL tube. The adherent cells were washed once with 2 mL calcium- and magnesium-free phosphate-buffered saline solution (1×, pH 7.0–7.2) (PBS) (GE Healthcare Life Sciences) to collect any remaining loosely adhering cells, and the wash was added to the 15-mL tube. Adherent cells were then lifted from their plate by trypsinization using 0.25% trypsin-EDTA (Gibco), and transferred to the 15-mL tube. The culture vessel was then washed once with 2 mL PBS, and the wash was added to the 15-mL tube. All collected floating and released adherent cells were then pelleted by centrifugation at 500 × *g* for 5 min and resuspended in fresh drug-free medium. Total cells were counted under phase-contrast using a haemocytometer. After cell counting, a recovery assay based on the original assay developed by Valiathan *et al.*^30^ was performed as follows. The cells were re-seeded post-treatment in a clear 12-well flat-bottom tissue culture plate (Corning) at a density of 30,000 cells per well in 1 mL drug-free medium. The plate was incubated for 2 h as described above. Then, culture confluence (*i.e*., cell surface area coverage) was measured immediately (0 h) and every 8 h thereafter using the IncuCyte S3 Live-Cell Analysis System (Sartorius). Thirty-six fields of view were imaged per well under phase-contrast at 10 × magnification using the standard scan type, and the collected images were analysed using the integrated confluence algorithm of the IncuCyte S3 2018C software (Sartorius). For each time point, the calculated culture confluence was averaged across the 36 fields of view, and normalized to the average culture confluence at 0 h.

### RNA disruption assays

Cells were seeded in a 6-well flat-bottom tissue culture plate at a density of 150,000 cells per well in 3 mL drug-free medium. The plate was incubated in a humidified incubator at 37 °C with 5% CO_2_ for 24 h. After 24 h, the medium was replaced with 3 mL fresh drug-free medium or fresh medium supplemented with ≤ 945 µM cycloheximide or ≤ 100 µM doxorubicin. In some experiments, 5 μM CsA (Sigma-Aldrich) or 100 nM TAR (Selleck Chemicals) were added to the medium (with or without doxorubicin) to inhibit ATP-binding cassette drug efflux transporter activity. The plate was incubated for 72 h as described above, and then the medium (containing the floating cells) was transferred to a 15-mL tube. The adherent cells were washed once with 2 mL PBS, and the wash was added to the 15-mL tube. The floating cells were pelleted by centrifugation at 234 × *g* for 5 min, resuspended in 5 mL PBS, and pelleted a second time. Then, 350 μL Buffer RLT (Qiagen) supplemented with 142 mM β-mercaptoethanol (Aldrich) were added directly to the wells to lyse the adherent cells with the aid of a cell lifter. The adherent cell lysate was mixed with the floating cell pellet, and homogenized by passing the lysate though a 21-gauge needle six times. Total RNA was isolated from the cell lysate using the RNeasy Mini kit (Qiagen), following the manufacturer’s protocol entitled ‘Purification of Total RNA from Animal Cells using Spin Technology’. The RNA preparation was heat-denatured at 70 °C for 3 min, and then the RNA quality was assessed by capillary gel electrophoresis using the 2100 Bioanalyzer (Agilent Technologies) with the RNA 6000 Nano kit (Agilent Technologies). The RDI was calculated for each sample using the electropherogram data and a proprietary algorithm developed by Rna Diagnostics, Inc.

### Clonogenic assays

Clonogenic assays were performed following a protocol adapted from Guo *et al.*^31^. Briefly, cells were seeded in a 6-well flat-bottom tissue culture plate at a density of 150,000 cells per well in 3 mL drug-free medium. The plate was incubated in a humidified incubator at 37 °C with 5% CO_2_ for 24 h. After 24 h, the medium was replaced with 3 mL fresh drug-free medium or fresh medium supplemented with either ≤ 945 µM cycloheximide or ≤ 100 µM doxorubicin. In some experiments, 5 μM CsA or 100 nM TAR were added to the medium (with or without doxorubicin). The plate was incubated for 24 h as described above, and then floating and adherent cells were collected and pelleted by centrifugation at 500 × *g* for 10 min as described for the cell counting and recovery assays. The pellet was washed once with 3 mL fresh drug-free medium. The cells were pelleted again by centrifugation at 500 × *g* for 10 min, and resuspended in 300 μL fresh drug-free medium. The cell suspension was then mixed with 2.7 mL drug-free semi-solid methylcellulose medium (comprising 1.96%_w/v_ methylcellulose [EMD], 0.7 × Iscove’s Modified Dulbecco’s Medium [Caisson Labs], 30%_v/v_ FBS, 35 U mL^−1^ penicillin, and 35 μg mL^−1^ streptomycin [GE Healthcare Life Sciences]) and incubated at 37 °C for 30 min. Then, 1.2 mL semi-solid cell mixture were added to a 6-well flat-bottom tissue culture plate using a 3-mL syringe equipped with a 16-gauge needle. The plate was incubated in a humidified incubator at 37 °C with 5% CO_2_ for 8 days, and colonies were counted using the IncuCyte S3 Live-Cell Analysis System and the IncuCyte S3 2018C software. Seven fields of view were imaged per well at 10 × magnification using the spheroid scan type. Colony counts were averaged across the seven fields of view for each treatment. In some experiments, the survival fraction was calculated as follows. For each individual experimental replicate, the number of colonies derived from drug-treated cells was divided by the number of colonies derived from untreated cells. The survival fraction was plotted against the log_10_-transformed drug concentration, and a three-parameter sigmoidal dose-response curve was fitted to the data points using Prism version 5.04 (GraphPad Software). The drug concentration at which the survival fraction was reduced by 50% (IC50) was then determined by interpolation from the regression curve.

### CCK8 assays

Cells were seeded in a clear 96-well flat-bottom tissue culture plate (Corning) at a density of 6,000 cells per well in 100 µL drug-free medium. The plate was incubated in a humidified incubator at 37 °C with 5% CO_2_ for 24 h. After 24 h, the medium was replaced with 100 μL fresh drug-free medium or fresh medium supplemented with ≤ 945 µM cycloheximide. To measure background absorbance, fresh media supplemented with various cycloheximide concentrations were added to empty wells. The plate was incubated for an additional 72 h as described above. The drug-containing medium was then replaced with 100 μL drug-free medium containing 10 µL CCK8 reagent (Dojindo Molecular Technologies), and the plate was incubated for 4 h as described above. The absorbance of the coloured formazan product, generated by bioreduction of the assay substrate, was measured at 450 nm using a Synergy H4 microplate reader (BioTek Instruments). Cell-free fresh media supplemented with and without CCK8 reagent were included in each run as negative controls.

### Trypan blue exclusion assays

Cells were seeded in a 6-well flat-bottom tissue culture plate at a density of 150,000 cells per well in 3 mL drug-free medium. The plate was incubated in a humidified incubator at 37 °C with 5% CO_2_ for 24 h. After 24 h, the medium was replaced with 3 mL fresh drug-free medium or fresh medium supplemented with ≤ 945 µM cycloheximide, and the plate was incubated for an additional 72 h as described above. Floating and adherent cells were then collected and resuspended as described for the cell counting and recovery assays. The cell suspension was diluted 1:2 with 0.4% Trypan blue solution (Sigma-Aldrich), and cells excluding the blue dye (*i.e*., cells with an intact plasma membrane) were counted using a haemocytometer.

### Quantification of P-gp expression

Cells were seeded in a 6-cm flat-bottom tissue culture plate (Sarstedt) at a density of 200,000 cells per plate in 3 mL drug-free medium. The plate was incubated in a humidified incubator at 37 °C with 5% CO_2_ for 24 h. After 24 h, the spent medium was discarded. The adherent cells were then washed twice with PBS and trypsinized. Cells were collected from the culture vessel in 5 mL PBS, and pelleted by centrifugation at 500 × *g* for 5 min. The cell pellet was subsequently resuspended in 80 µL stain buffer (2% FBS in PBS) and either 20 µL R-phycoerythrin-conjugated mouse anti-human P-gp antibody (Clone 17F9, BD Pharmingen, BD Biosciences) or 20 µL R-phycoerythrin-conjugated mouse IgG2b κ isotype control antibody (Clone 27–35, BD Pharmingen, BD Biosciences). After a 30-min incubation in the dark at room temperature, 1 mL PBS was added to each cell-antibody mixture. The cells were then pelleted by centrifugation at 500 × *g* for 5 min, resuspended in 500 μL PBS, and loaded onto an FC500 flow cytometer (Beckman Coulter). Samples were analysed under channel FL3 using an excitation wavelength of 488 nm and an emission wavelength of 585 ± 15 nm. Analyses were stopped at 20,000 events or after 300 s. The results were captured and analysed using the CXP Analysis software version 2.2 (Beckman Coulter), with all samples being ungated.

### Quantification of intracellular doxorubicin

Cells were seeded in a 6-cm flat-bottom tissue culture plate at a density of 200,000 cells per plate in 3 mL drug-free medium. The plate was incubated in a humidified incubator at 37 °C with 5% CO_2_ for 24 h. After 24 h, the medium was replaced with 3 mL fresh drug-free medium or fresh medium supplemented with 500 nM doxorubicin. In some experiments, 5 μM CsA or 100 nM TAR were added to the medium (with or without doxorubicin). The plate was incubated for an additional 24 h as described above. Floating and adherent cells were collected and pelleted as described for the cell counting and recovery assays. The supernatant was carefully decanted and discarded, and the 15-mL centrifuge tube was placed upside-down on a piece of tissue paper to collect any residual liquid. The cell pellet was resuspended in 500 μL PBS, and loaded onto an FC500 flow cytometer. Samples were analysed as described for P-gp quantification, using channel FL4.

### Statistical analyses

All statistical analyses were performed using R version 3.6.0^32^ and RStudio version 1.1.423^33^. To determine the effect of cycloheximide on (i) total cell counts, (ii) cell recovery, (iii) RNA disruption, (iv) colony formation, (v) cellular dehydrogenase activity, and (vi) plasma membrane integrity, Kruskal-Wallis rank sum tests were performed using the *kruskal.test* function from the *stats* package^32^. The distributions of the observations in each group did not have a similar shape and/or spread, as determined by visually inspecting the plotted data and by conducting the Fligner-Killeen test using the *fligner.test* function from the *stats* package^32^; the Kruskal-Wallis tests were therefore used as tests of stochastic dominance. Drug concentrations significantly decreasing total cell counts, cell recovery, colony formation, cellular dehydrogenase activity and membrane integrity, and concentrations significantly increasing RNA disruption in ovarian cancer cells, were then identified using Conover’s one-tailed many-to-one rank comparison test with the *kwManyOneConoverTest* function from the *PMCMRplus* package^34^. *P* values were adjusted for multiple comparisons using Holm’s method with the function’s *p.adjust.method* option.

To evaluate the effect of drug resistance and drug/inhibitor treatment on (i) RNA disruption, (ii) intracellular doxorubicin levels, and (iii) colony formation, and to determine the effect of drug resistance and the selected antibody on detected P-gp expression levels, two-way analyses of variance (ANOVAs) with type III sums of squares were performed using the *Anova* function from the *car* package^35^. The data sets were evaluated for their conformity with the assumptions of normality and homoscedasticity. Each model’s residuals were confirmed to be normally distributed by visually inspecting a normal quantile-quantile plot and by performing the Shapiro-Wilk test using the *shapiro.test* function from the *stats* package^32^. The data was also verified to be homoscedastic by visually inspecting a residual vs. fitted value plot and by conducting either the Bartlett test or Levene’s Median test using the *bartlett.test* function from the *stats* package^32^ or the *leveneTest* function from the *car* package^35^, respectively. The measurement values were ln-transformed in order to meet the assumptions of the test. *Post-hoc* multiple comparisons of least-squares means were then carried out using the *lsmeans* function from the *lsmeans* package^36^. *P* values were adjusted for multiple comparisons using the Tukey method with the function’s *adjust* option.

For all statistical analyses, *P* ≤ 0.05 was deemed significant. Unless otherwise indicated, untransformed data are presented in the figures.

### Graphics

Electropherograms were generated by the 2100 Expert software version B.02.09.SI725 (SR1) (Agilent Technologies). Flow cytometry graphs were generated by the CXP Analysis software version 2.2. Graphs were prepared using either Prism version 5.04 or R version 3.6.0^32^ with RStudio version 1.1.423^33^. Figures were assembled and annotated using PowerPoint for Mac 2011 version 14.7.7 (Microsoft Corporation).

## Supplementary information

Supplementary Information.

## Data Availability

All data collected and analysed during this study are provided in the manuscript or in the accompanying Supplementary Information document.
