# Supplementary material for: The RNA disruption assay is superior to conventional drug sensitivity assays in detecting cytotoxic drugs
Source: Sci Rep. 2020 May 26;10:8671. doi: 10.1038/s41598-020-65579-9 (PMC7250890; doi:10.1038/s41598-020-65579-9)
Supplement: Supplementary file 1 — Supplementary Information. [file 41598_2020_65579_MOESM1_ESM.pdf]

*Scientific Reports*

# Supplementary Information

## The RNA disruption assay is superior to conventional drug sensitivity assays in detecting cytotoxic drugs

Jonathan P. J. Mapletoft[1], Renée J. St-Onge[2], Baoqing Guo[3], Phillipe Butler[1], Twinkle J. Masilamani[2], Lavina D'costa[2], Laura B. Pritzker[2], Amadeo M. Parissenti[1, 2, 3, 4] *

[1] Graduate Program in Chemical Sciences, Laurentian University, Sudbury, ON, Canada

[2] Rna Diagnostics, Inc., Sudbury and Toronto, ON, Canada

[3] Health Sciences North Research Institute, Sudbury, ON, Canada

[4] Division of Medical Sciences, Northern Ontario School of Medicine, Sudbury, ON, Canada

*** Corresponding author:** Amadeo M. Parissenti, telephone: 1-705-522-6237, e-mail: aparissenti@hsnri.ca

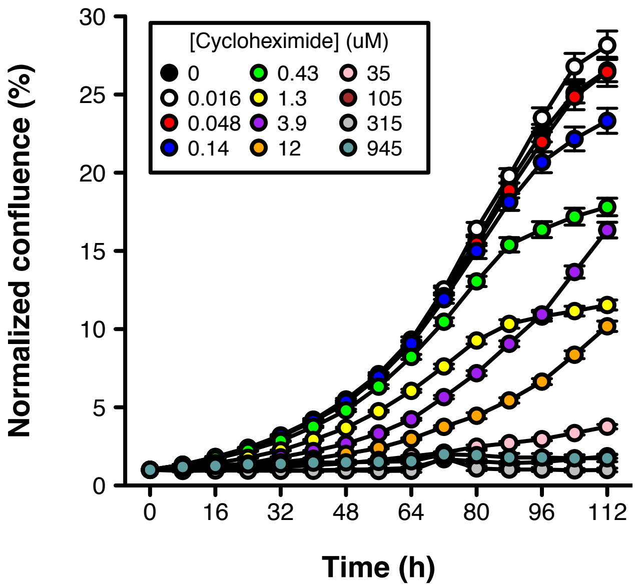


**Supplementary Figure S1.** Recovery of ovarian cancer cells following cycloheximide treatment. A2780 cells were treated for 72 h with 0-945 µM cycloheximide. Cells were then collected, resuspended in drug-free medium, and seeded into plates. Culture confluence was measured every 8 h for a total of 112 h, and normalized to the initial confluence. Data are presented as means of 36 technical replicates ± standard error. The image is representative of three independent biological replicates.

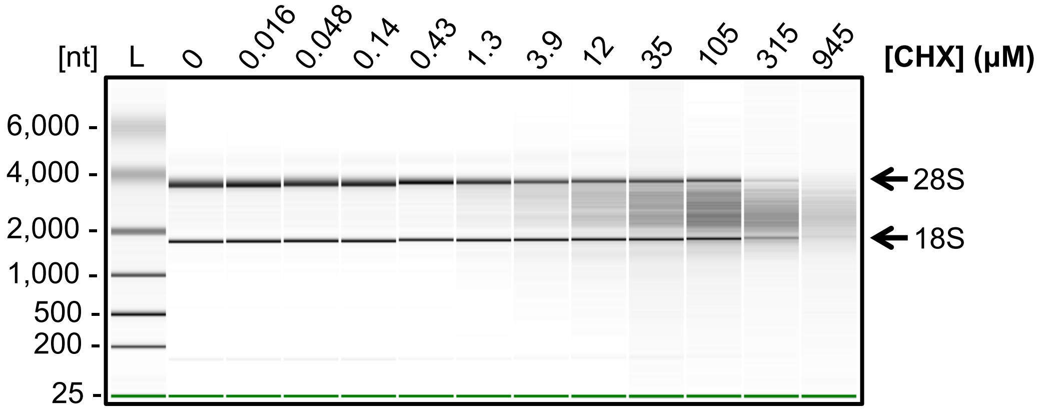


**Supplementary Figure S2.** Cycloheximide-induced RNA disruption in ovarian cancer cells. Total RNA was isolated from A2780 cells treated for 72 h with 0-945 µM cycloheximide (CHX), and size-separated by capillary gel electrophoresis. Full-length 18S and 28S rRNA bands are indicated with arrows. The electropherogram is representative of three independent biological replicates. L, RNA 6000 Ladder (Agilent Technologies), in nucleotides (nt).

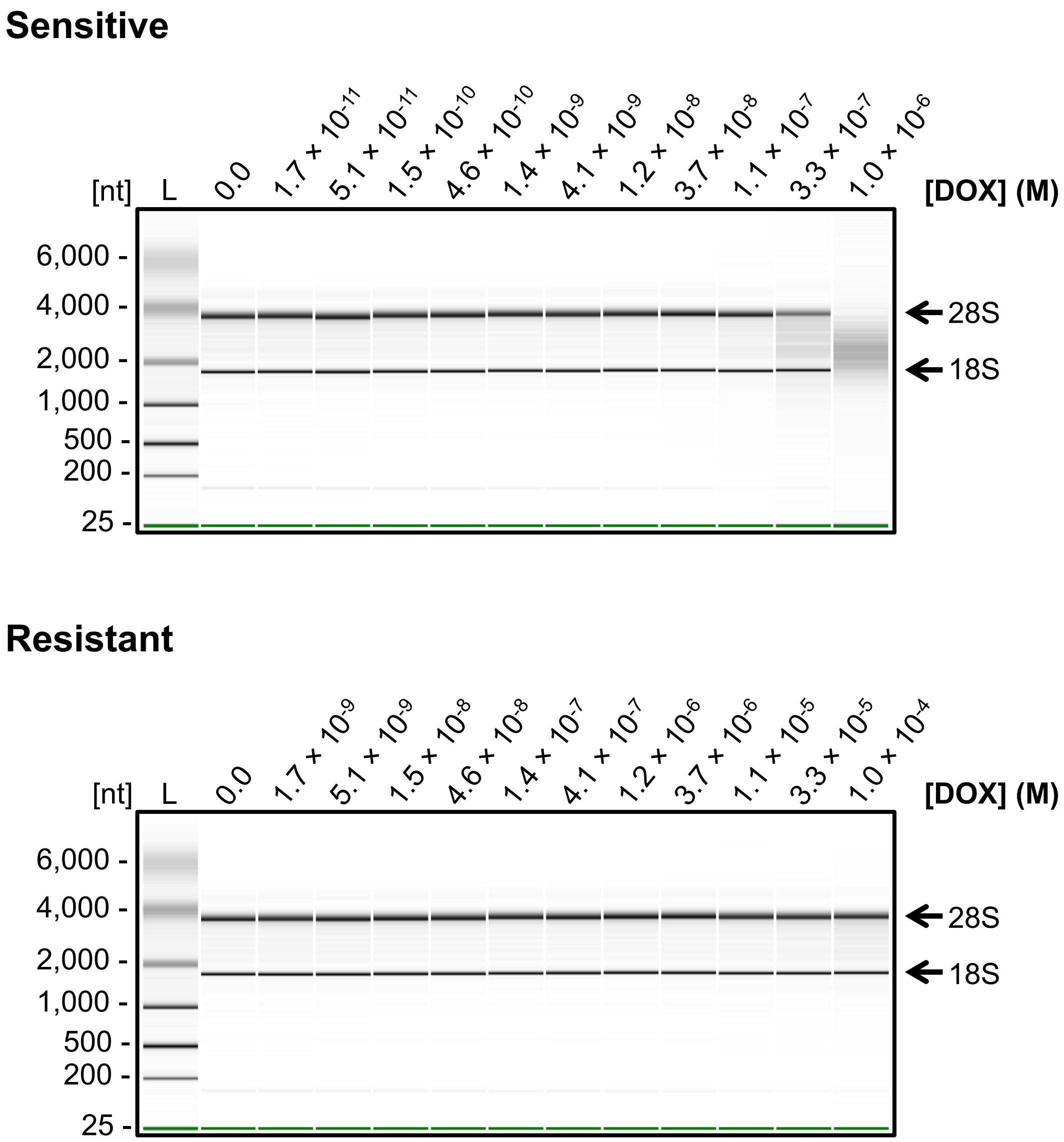


**Supplementary Figure S3.** Doxorubicin-induced RNA disruption in drug-sensitive and drug-resistant ovarian cancer cells. Doxorubicin-sensitive A2780 cells (Sensitive) and doxorubicin-resistant $A2780_{ADR}$ cells (Resistant) were treated for 72 h with 0-1 μM and 0-100 μM doxorubicin (DOX), respectively. Total RNA was extracted from cells, and size-separated by capillary gel electrophoresis. Full-length 18S and 28S rRNA bands are indicated with arrows. The electropherograms are representative of four independent biological replicates. L, RNA 6000 Ladder (Agilent Technologies), in nucleotides (nt).

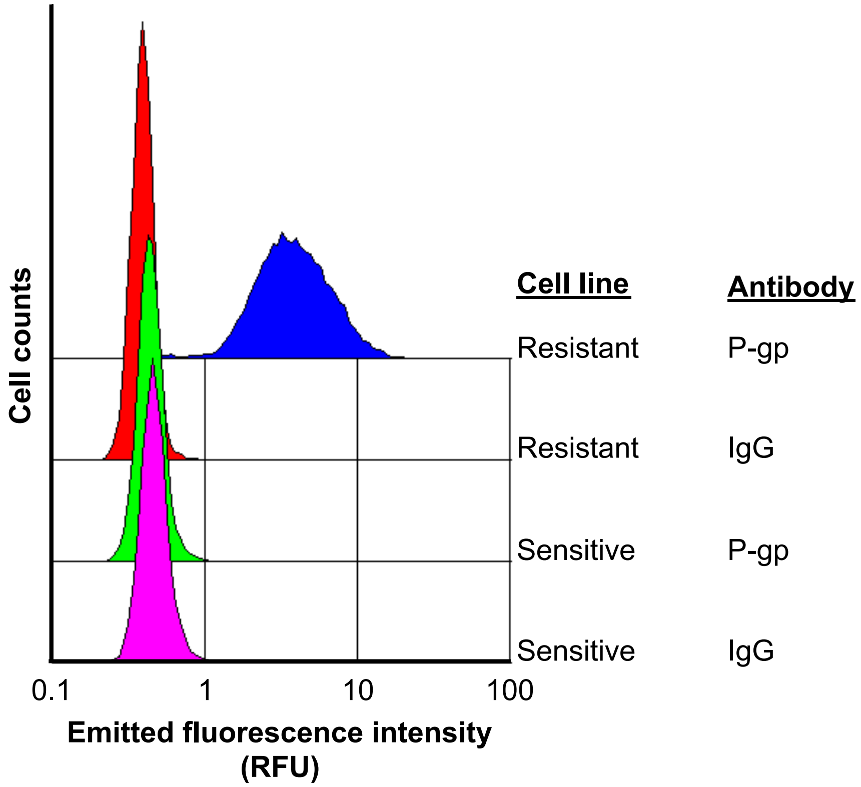


**Supplementary Figure S4** Cell surface-associated P-gp expression in doxorubicin-sensitive and -resistant ovarian cancer cells. Surface expression of P-gp in doxorubicin-sensitive A2780 cells (Sensitive) and doxorubicin-resistant $A2780_{ADR}$ cells (Resistant) was assessed by flow cytometry using an R-phycoerythrin-conjugated mouse anti-human P-gp antibody (P-gp). Flow cytometry using an R-phycoerythrin-conjugated mouse IgG2b κ isotype antibody (IgG) was also performed in parallel, as a negative control. The image is representative of four independent biological replicates. RFU, relative fluorescence units.

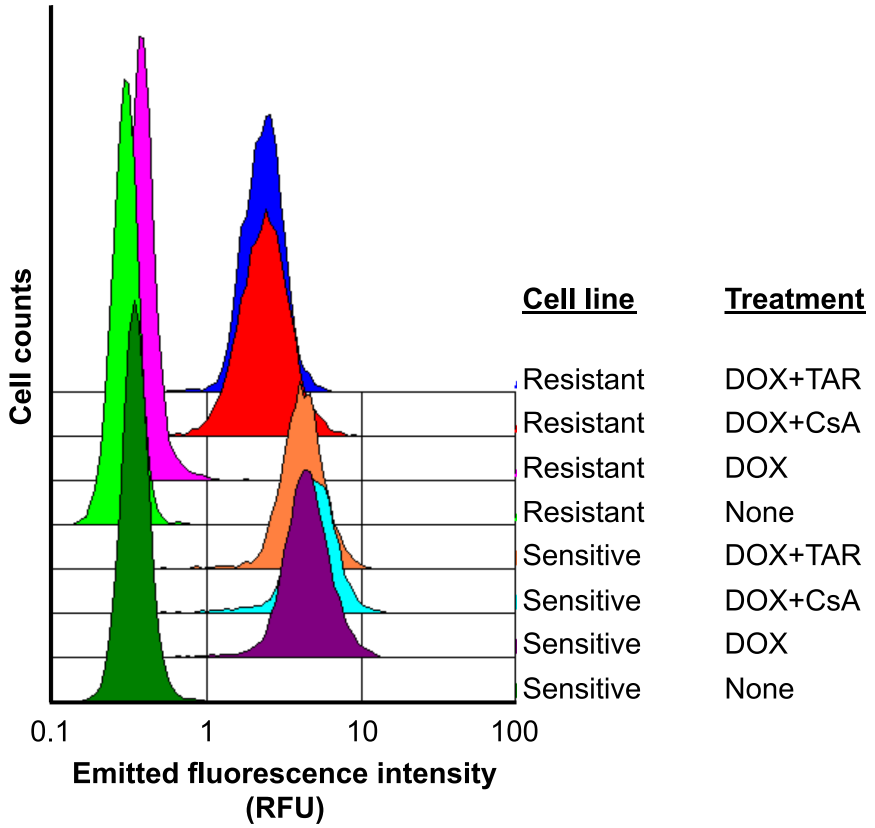


**Supplementary Figure S5.** Effect of P-gp inhibition on intracellular drug accumulation in drug-treated doxorubicin-sensitive and -resistant ovarian cancer cells. Doxorubicin-sensitive A2780 cells (Sensitive) and doxorubicin-resistant A2780$_{ADR}$ cells (Resistant) were treated for 24 h with 0 or 500 nM doxorubicin (DOX), in the presence or absence of 5 μM CsA or 100 nM TAR. Intracellular doxorubicin levels were then quantified by flow cytometry. The image is representative of three independent biological replicates. RFU, relative fluorescence units.

## 0.5 µM doxorubicin

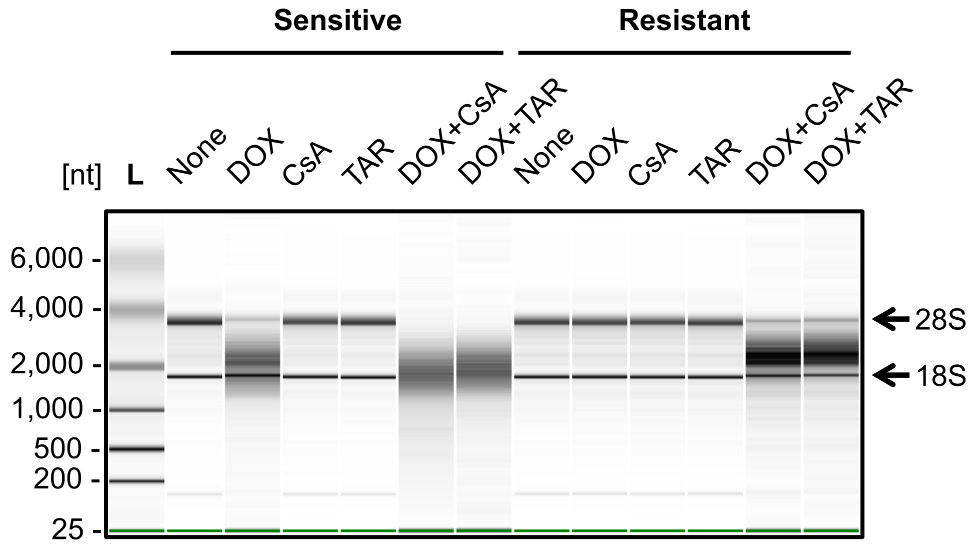


## 1 µM doxorubicin

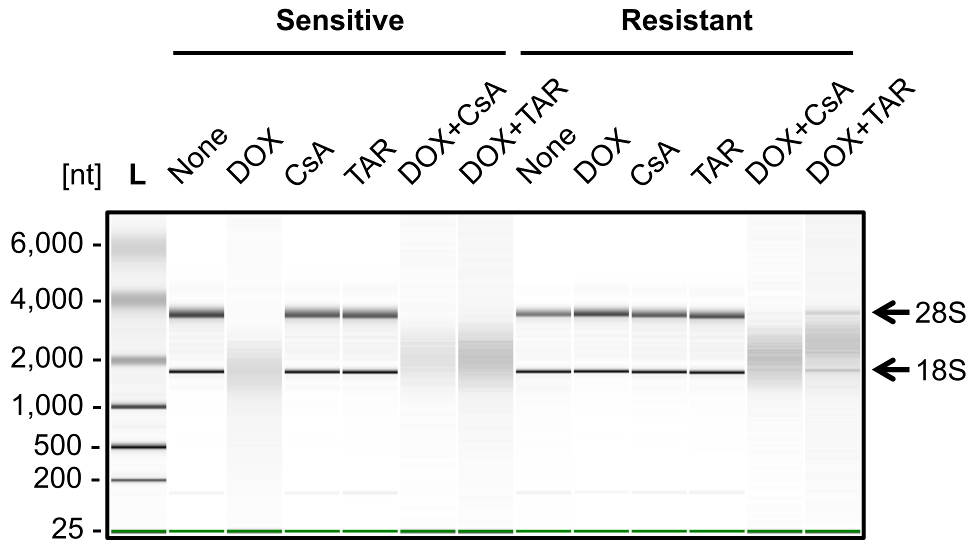


**Supplementary Figure S6.** Effect of P-gp inhibition on RNA disruption in drug-treated doxorubicin-sensitive and -resistant ovarian cancer cells. Doxorubicin-sensitive A2780 cells (Sensitive) and doxorubicin-resistant $A2780_{ADR}$ cells (Resistant) were treated for 72 h with 0, 0.5 or 1 µM doxorubicin (DOX), in the presence or absence of 5 µM CsA or 100 nM TAR. Total RNA was extracted from cells, and size-separated by capillary gel electrophoresis. Full-length 18S and 28S rRNA bands are indicated with arrows. The electropherograms are representative of four (0.5 µM doxorubicin) or three (1 µM doxorubicin) independent biological replicates. L, RNA 6000 Ladder (Agilent Technologies), in nucleotides (nt).

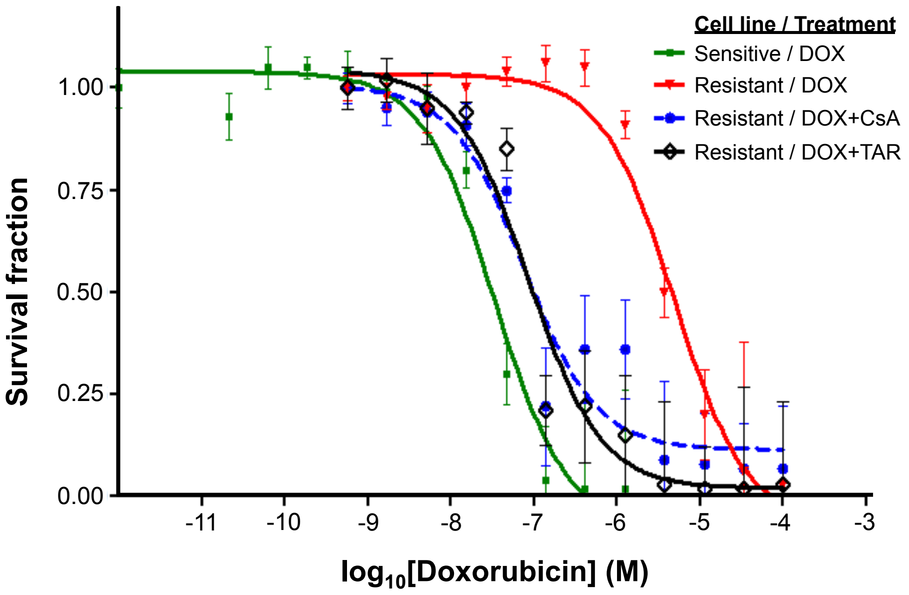


**Supplementary Figure S7.** Effect of P-gp inhibition on colony formation by drug-treated doxorubicin-sensitive and -resistant ovarian cancer cells. Doxorubicin-sensitive A2780 cells (Sensitive) and doxorubicin-resistant $A2780_{ADR}$ cells (Resistant) were treated for 24 h with a wide range of doxorubicin (DOX) concentrations, in the absence or in the presence of either 5 μM CsA or 100 nM TAR. Proliferating cells remaining post-treatment were then counted using the clonogenic assay. The data are presented as means of 7 technical replicates ± standard error. The image is representative of three biological replicates.

**Supplementary Table S1.** Comparison between the RDA and other drug sensitivity assays.

| Assay | Sensitivity parameter | Discrimination between viable and dead cells | Labour intensity | Automation | Assay time | Cost[b] |
|---|---|---|---|---|---|---|
| RDA | RNA disruption | Excellent | Intermediate | Possible | Less than 1 day | $$$ |
| Clonogenic assay | Cell division | Good | Intermediate to high[a] | Difficult[a] | 1-3 weeks | $$$ |
| CCK8 assay | Cellular dehydrogenase activity | Poor | Low | Possible | Less than 1 day | $$ |
| Trypan blue exclusion assay | Membrane integrity | Poor | Low | Possible | Less than 1 day | $ |

[a] Colonies can be counted manually or using a system similar to the IncuCyte S3 Live-Cell Analysis System. Automated counting reduces the labour intensity of the assay.
[b] The relative cost of each assay is depicted by an increasing number of dollar signs.
